# Burst, Short, and Sustained Vitamin D_3_ Applications Differentially Affect Osteogenic Differentiation of Human Adipose Stem Cells

**DOI:** 10.3390/ijms21093202

**Published:** 2020-04-30

**Authors:** Cindy Kelder, Jolanda M.A. Hogervorst, Daniël Wismeijer, Cornelis J. Kleverlaan, Teun J. de Vries, Astrid D. Bakker

**Affiliations:** 1 Department of Oral Implantology and Prosthodontics, Academic Centre For Dentistry Amsterdam (ACTA), University of Amsterdam and Vrije Universiteit Amsterdam, Gustav Mahlerlaan 3004, 1081 LA Amsterdam, The Netherlands; c.l.kelder@acta.nl (C.K.); d.wismeijer@acta.nl (D.W.); 2 Department of Oral Cell Biology, Academic Centre For Dentistry Amsterdam (ACTA), University of Amsterdam and Vrije Universiteit Amsterdam, Amsterdam Movement Sciences, Gustav Mahlerlaan 3004, 1081 LA Amsterdam, The Netherlands; jma.hogervorst@acta.nl; 3 Department of Dental Material Sciences, Academic Centre For Dentistry Amsterdam (ACTA), University of Amsterdam and Vrije Universiteit Amsterdam, Gustav Mahlerlaan 3004, 1081 LA Amsterdam, The Netherlands; c.kleverlaan@acta.nl; 4 Department of Periodontology, Academic Centre For Dentistry Amsterdam (ACTA), University of Amsterdam and Vrije Universiteit Amsterdam, Amsterdam Movement Sciences, Gustav Mahlerlaan 3004, 1081 LA Amsterdam, The Netherlands; teun.devries@acta.nl

**Keywords:** sustained release, burst release, short stimulation, bone, bioactive components, calcitriol, tissue engineering

## Abstract

Incorporation of 1,25(OH)_2_ vitamin D_3_ (vitD_3_) into tissue-engineered scaffolds could aid the healing of critical-sized bone defects. We hypothesize that shorter applications of vitD_3_ lead to more osteogenic differentiation of mesenchymal stem cells (MSCs) than a sustained application. To test this, release from a scaffold was mimicked by exposing MSCs to exactly controlled vitD_3_ regimens. Human adipose stem cells (hASCs) were seeded onto calcium phosphate particles, cultured for 20 days, and treated with 124 ng vitD_3_, either provided during 30 min before seeding ([200 nM]), during the first two days ([100 nM]), or during 20 days ([10 nM]). Alternatively, hASCs were treated for two days with 6.2 ng vitD_3_ ([10 nM]). hASCs attached to the calcium phosphate particles and were viable (~75%). Cell number was not affected by the various vitD_3_ applications. VitD_3_ (124 ng) applied over 20 days increased cellular alkaline phosphatase activity at Days 7 and 20, reduced expression of the early osteogenic marker *RUNX2* at Day 20, and strongly upregulated expression of the vitD_3_ inactivating enzyme *CYP24*. VitD_3_ (124 ng) also reduced *RUNX2* and increased *CYP24* applied at [100 nM] for two days, but not at [200 nM] for 30 min. These results show that 20-day application of vitD_3_ has more effect on hASCs than the same total amount applied in a shorter time span.

## 1. Introduction

Bone defects can occur as a consequence of birth defects, injury, cancer, or inflammation. Despite the good intrinsic healing capacity of bone, the reconstruction of critical size defects remains challenging. Currently, the “golden standard” for critical size bone defects is the use of autologous bone grafts [1]. These grafts are taken from the patient, often from the iliac crest, or in the case of orofacial defects from the symphysis of the chin or ascending ramus. Autologous bone is generally still considered the “gold standard” since it provides both an osteogenic and osteoconductive environment, due to the presence of live cells and the cocktail of growth factors present in the matrix [2]. However, this method suffers from two major drawbacks: donor site morbidity and lack of available tissue [3]. These drawbacks could be overcome by alternative approaches for bone reconstruction, such as methods employing principles of bone tissue engineering.

To regenerate bone, bone tissue engineering combines the use of biomaterials, e.g., polymer-based or calcium phosphate-based materials, cells, and physical, mechanical, and/or chemical stimuli [4], such as bone morphogenetic protein-2 (BMP-2) and vascular endothelial growth factor (VEGF) [5,6]. Another biological component used as a chemical stimulus in bone regeneration is 1,25(OH)_2_ vitamin D_3_ (vitD_3_), which promotes osteogenic differentiation of bone marrow-derived mesenchymal stem cells (BMSCs) and hASCs by upregulation of alkaline phosphatase (ALP) and enhancing mineralization [7,8,9]. 

For the delivery of biological components, for example, BMP-2 and vitD_3_, multiple delivery methods can be considered. Here, we focus on the three main delivery methods. First, an injection of bioactive factors into a carrier or injured site, or adsorption to a surface. This leads to a burst release where the biological component is depleted after a couple of days, as has been described for BMP-2 [10]. Supra-physiological concentrations are often used in this case, which may lead to negative effects such as a growth-inhibition when vitD_3_ is used [11,12] or ectopic bone formation for BMP-2 [13]. Furthermore, in the case of expensive biological components such as BMP-2, this method can become costly. As a second method of release, avoiding supra-physiological concentrations, biomaterials can be used as release vehicles allowing a more sustained release [14,15,16]. A drawback of longer stimulation with biological components is that it can lead to the formation of inhibitors. For example, BMP-2 induces the expression of its antagonist noggin [17,18] and vitD_3_ induces the expression of Cytochrome P450 family 24 subfamily A member 1 (CYP24a1), an enzyme that inactivates vitD_3_ [19,20]. In addition, in natural bone development or healing, cytokines, growth factors, or extracellular matrix components are usually transiently expressed [21,22]. Sustained expression of these factors hinders progression of healing or development [23]. The third method is a short stimulation of stem cells with a bioactive factor (30 min or less) of cells, after which the biological component is washed away [24]. Mokhtari-Jafari et al. showed that such a short stimulation with vitD_3_ can lead to the differentiation of adipose stem cells into the osteogenic lineage [25]. Short incubation (15 or 30 min) with growth factors or other bioactive compounds suffices to induce long lasting (weeks) effects on hASCs in cell culture [23,24]. In some cases, these effects even become more pronounced over time. To give an example, short incubation of BMP-2 triggers expression of the late marker osteocalcin in hASCs after 21 days of culture, but not at earlier time points [23]. Short stimulation can be used in a one-step surgery where the cells are harvested, stimulated shortly, and combined with a biomaterial serving as scaffold, which can then be implanted, in one procedure. In this way, no exogenous ligands are placed into the body, avoiding possible side effects.

**Figure 1:**
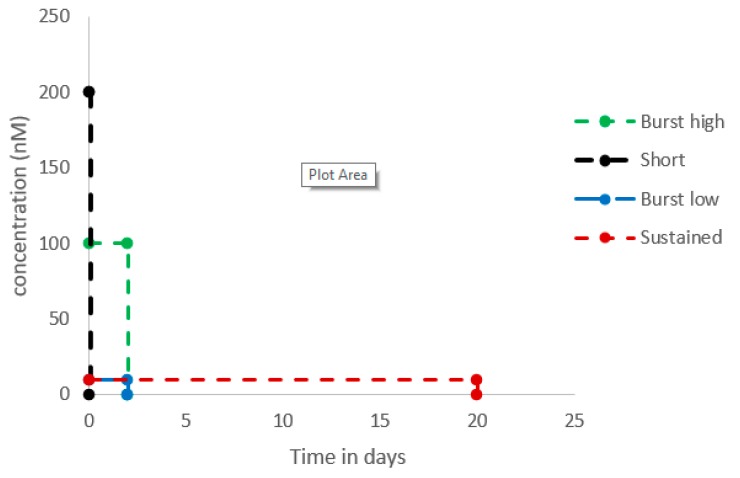
Schematic representation of the different vitamin D_3_ applications. A total of 124 ng of vitD_3_ was added for either 30 min before seeding at a concentration of 200 nM (Short), for two days at a concentration of 100 nM (Burst high) to mimic a burst release, or for 20 days with a concentration of 10 nM (Sustained) to mimic a sustained release. Alternatively, cells were exposed to 10 nM vitD_3_ for two days (Burst low).

To shed light on the ongoing discussion whether short, burst or sustained release is the best method for the application of biological factors for bone tissue engineering, we designed an experiment where the in vivo situation was mimicked in a cell culture environment of hASCs seeded on BCP particles, allowing precise manipulation of doses and timing of application of a biologically active factor. We used vitD_3_, since we successfully used this factor to stimulate osteogenic differentiation of hASCs in conventional 2D cultures. Our aim was to investigate which application of vitD_3_, i.e., mimicking short exposure of cells ([200 nM] for 30 min), burst release from scaffolds ([100 nM] for two days), or sustained release from scaffolds ([10 nM] for 20 days), leads to optimal osteogenic differentiation of hASCs (Figure 1). We hypothesized that shorter applications of vitD_3_ are more beneficial for osteogenic differentiation than sustained application because longer applications may lead to the formation of inhibitors. 

## 2. Results

### 2.1. hASCs Attached to BCP Particles and Survived, in the Presence and Absence of Vitamin D_3_

**Figure 2:**
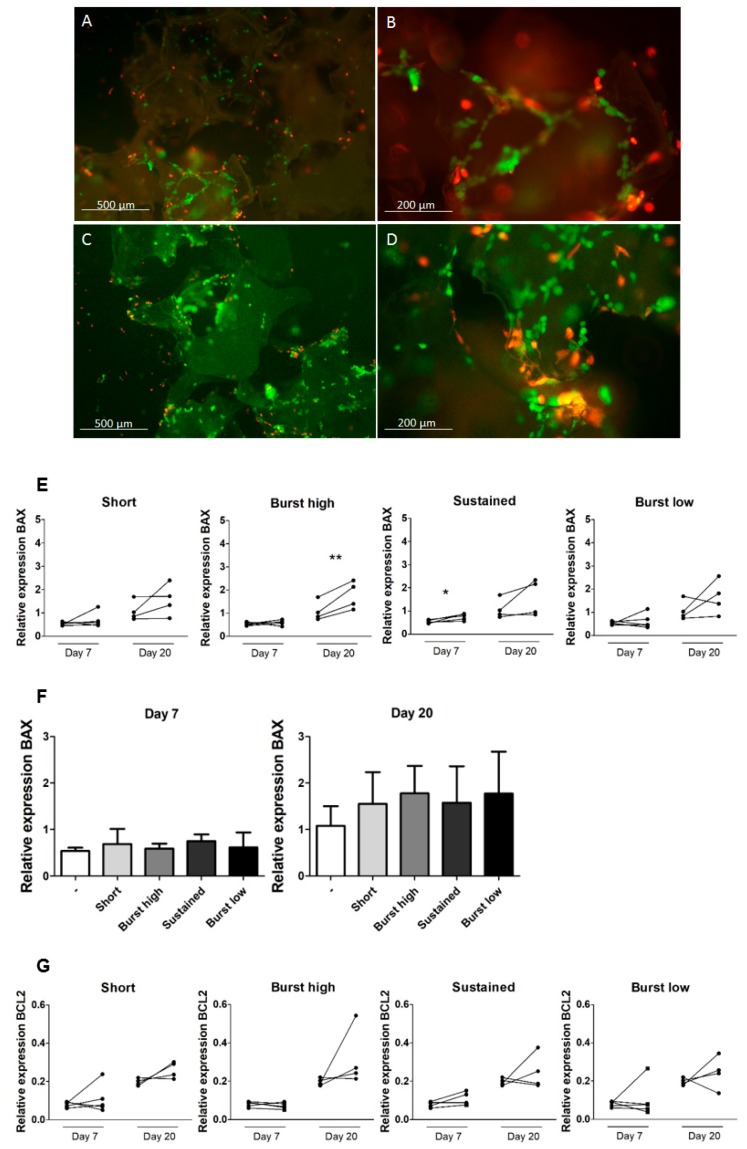
Effect of vitamin D_3_ on the viability of hASCs on BCP particles. Fluorescence microscopy of live/dead staining shows the attachment and viability of hASCs on Day 2 after seeding. Living cells are stained green, while the red-stained cells are dead/dying. (**A**,**B**) Sample without vitD_3_ treatment, where (B) is a higher magnification of the same sample as (A). (**C**,**D**) A representative sample with vitD_3_ treatment (highest concentration, i.e., [200 nM]) is depicted. Both (C,D) were made in the same sample. (**E**) Gene expression of pro-apoptotic marker *BAX* measured at Days 7 and 20. Gene expression in controls (left) and vitD_3_-treated cultures (right) of the same donor are connected by a line. (**F**) Relative expression of *BAX* at Days 7 and 20. Data represent mean + SD of four or five independent donors. (**G**) Gene expression of anti-apoptotic marker *BCL-2* measured at Days 7 and 20. Gene expression in controls (left) and vitD_3_-treated cultures (right) of the same donor are connected by a line. * Significant effect (*p* < 0.05) of vitD_3_ compared to the control at one time point. ** *p* < 0.01. All gene expression levels were normalized to *HPRT*.

Before identifying the optimal vitD_3_ application, it needs to be established whether the hASCs attached to the BCP particles. In Figure 2, representative micrographs for samples without vitD_3_ (Figure 2A,B) and with vitD_3_ (Figure 2C,D) are shown. The micrographs with vitD_3_ are from samples with short application (200 nM: 30 min) of vitD_3._ These micrographs are representative for all vitD_3_ -treated samples. By manual counting of green and red cells, it was determined that a comparable number of hASCs had attached to the BCP particles at Day 2, regardless of vitD_3_ treatment. In all vitD_3_ treated groups, as well as in the control group, the percentage viable (green) cells was approximately 75%. No exact quantification is given per group, as this would misrepresent the reliability with which these numbers can be established, since it is extremely difficult to reliably visualize and discern individual cells in 3D seeded particles (Figure 2A–D).

Apoptosis induced by vitD_3_ was measured by gene expression of pro-apoptotic protein B-cell lymphoma protein 2 associated X (*BAX*) and its inhibitor B-cell lymphoma 2 (*BCL-2*) (Figure 2E,F). The balance between *BCL-2* and *BAX* protein expression strongly affects apoptosis in bone cells, whereby an excess of *BCL-2* protects against apoptosis. In the current paper, *BAX* gene expression is therefore considered a pro-apoptotic marker, whereas enhanced BCL-2 gene expression is seen as a marker correlated with inhibition of apoptosis. At Day 7, Sustained application significantly increased the relative expression of *BAX* compared to the control and at Day 20. Burst high application of vitD_3_ significantly increased the expression of *BAX* at Day 20 compared to the control (Figure 2E). All other applications did not affect gene expression of this pro-apoptotic factor. Average *BAX* expression did not significantly differ between vitD_3_-treated groups (Figure 2F). None of the vitD_3_ treatments had an effect on the expression of *BCL-2* (Figure 2G). In general, the expression of *BCL-2* appeared to be upregulated at later time points compared to earlier time points within the same vitD_3_ treatment group, but this was not statistically tested (Figure 2G).

### 2.2. Vitamin D_3_ Did not Affect Proliferation of hASCS on BCP Particles

**Figure 3:**
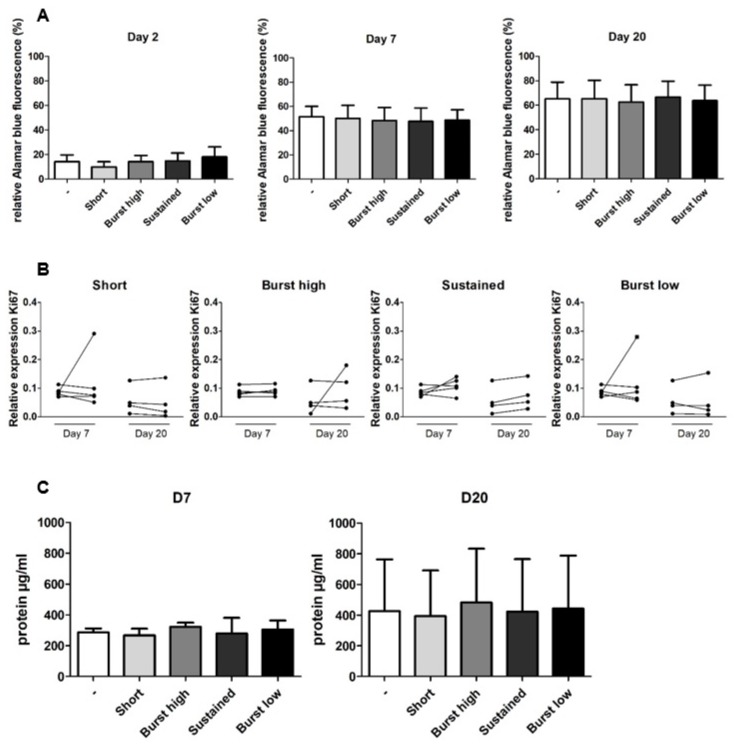
**** Effect of vitamin D_3_ on proliferation and protein content of hASCs. hASCs were subjected to the various vitD_3_ treatment modalities and Alamar blue was measured at Days 2, 7, and 20; the gene expression of *Ki67* was measured at Days 7 and 20; and total protein content was measured at Days 7 and 20. (**A**) VitD_3_ treatment did not affect Alamar blue measurement between any of the groups, at any time point measured. (**B**) Gene expression of proliferation marker *Ki67* did not significantly differ between vitD_3_-treated and control groups. Gene expression in controls (left) and vitD_3_-treated cultures (right) of the same donor are connected by a line. (**C**) Total protein content was similar between all vitD_3_-treated groups at Day 7, as well as on Day 20. Data represent four or five independent donors (mean + SD). Gene expression data was normalized to *HPRT*.

Having established that hASCs indeed attached to BCP particles and were viable in the early stage (Figure 2), we next assessed the effect of vitD_3_ on hASC proliferation. Alamar blue assay, gene expression of *Ki67*, and total protein levels were used as measures of cell number. Over time, between Days 2 and 20 the percentage of conversed Alamar blue, used as an indirect measure of cell number, increased on average 4.7-fold in all groups (Figure 3A). Compared to the control, VitD_3_ did not significantly affect the percentage of conversed Alamar blue for any mode of application, at any time point tested. The effect of the different vitD_3_-treatment kinetics on cell proliferation was also assessed by quantification of gene expression of the cell proliferation marker *Ki67*, which was not affected by vitD_3_ treatment (Figure 3B). For confirmation of cell number, total protein per condition was analyzed. In line with earlier results, total protein was comparable between the groups treated with and without vitD_3_ at Days 7 and 20 (Figure 3C). Between Days 7 and 20, total protein seemed to increase similarly for all groups, but this was not statistically tested.

### 2.3. Regime Mimicking Sustained Release of Vitamin D_3_ Affects ALP and RUNX2

**Figure 4:**
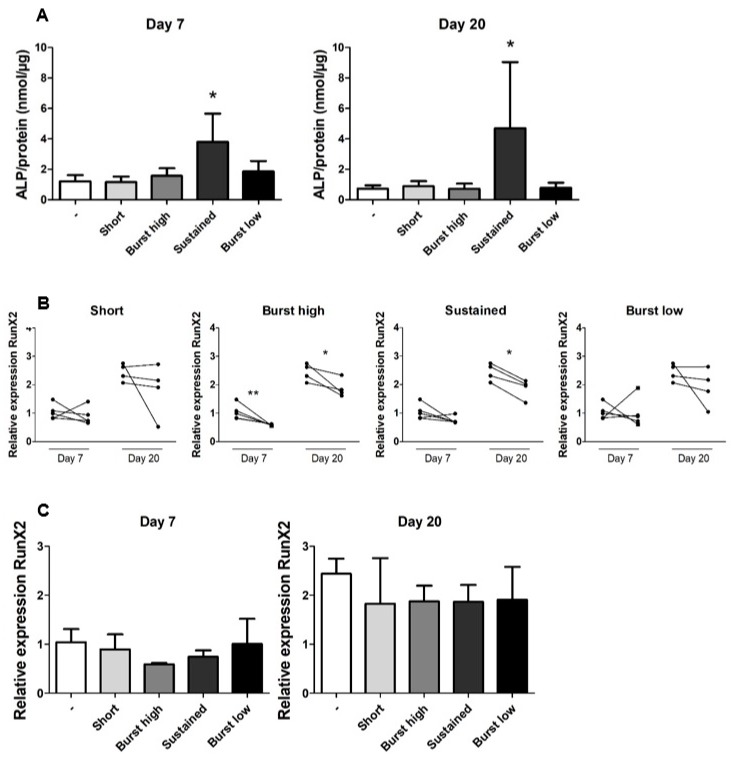
Effect of vitamin D_3_ on ALP activity and *RUNX2* expression. (**A**) Cellular ALP, as a measure for early osteogenic differentiation, was measured on Days 7 and 20. Sustained-release significantly increased ALP activity compared to all other groups at both time points. (**B**) *RUNX2* expression in general seemed to be higher at Day 20 compared to Day 7, although no statistical comparison was made. VitD_3_ seemed to reduce *RUNX2* expression in general. This effect of vitD_3_ was significant for burst high at Days 7 and 2, and for sustained application at Day 20. Gene expression in controls (left) and vitD_3_-treated cultures (right) of the same donor are connected by a line. (**C**) Average relative expression of *RUNX2* at Days 7 and 20. Data represent four or five independent donors (mean + SD). All gene expression levels were normalized to *HPRT*. * Significant difference (*p* < 0.05) between control and vitD_3_ treatment at the same time point ** *p* < 0.01.

The activity of ALP is an indication of early differentiation of the hASCs. ALP activity was measured on Days 7 and 20, and was divided by total protein (µg) levels to correct for approximate cell number. Sustained application of vitD_3_ significantly increased cellular ALP (Figure 4A). At Day 7, Sustained application increased the activity of ALP on average by 2.7-fold, compared to all the other conditions. On Day 20, this increase was 6.0-fold. Gene expression of runt-related transcription factor 2 (*RUNX2*), osteopontin (*OPN*) and collagen type 1a (*COL1a*) were measured as positive osteogenic differentiation markers. Adipogenic marker peroxisome proliferator-activated receptor gamma (*PPAR-γ*) was quantified as a negative marker for osteogenic differentiation. *RUNX2* is an early differentiation marker which in general appeared to be upregulated at Day 20 compared to Day 7 within the same vitD_3_ treatment group, but this was not statistically tested. The Burst high application at Days 7 and 20 and the Sustained vitD_3_ application at Day 20 significantly downregulated the expression of *RUNX2* compared to their (paired) untreated controls (Figure 4B). When the rather variable *RUNX2* expression levels were expressed as average, no significant difference in gene expression between the different vitD_3_-treated groups and the control was found (Figure 4C). VitD_3_ did not significantly affect *OPN*, *COL1a*, or *PPAR-γ* expression at any concentration, at any time point (Figure S1).

### 2.4. Sustained Application of Vitamin D_3_ Highly Upregulates CYP24a1 Expression

**Figure 5:**
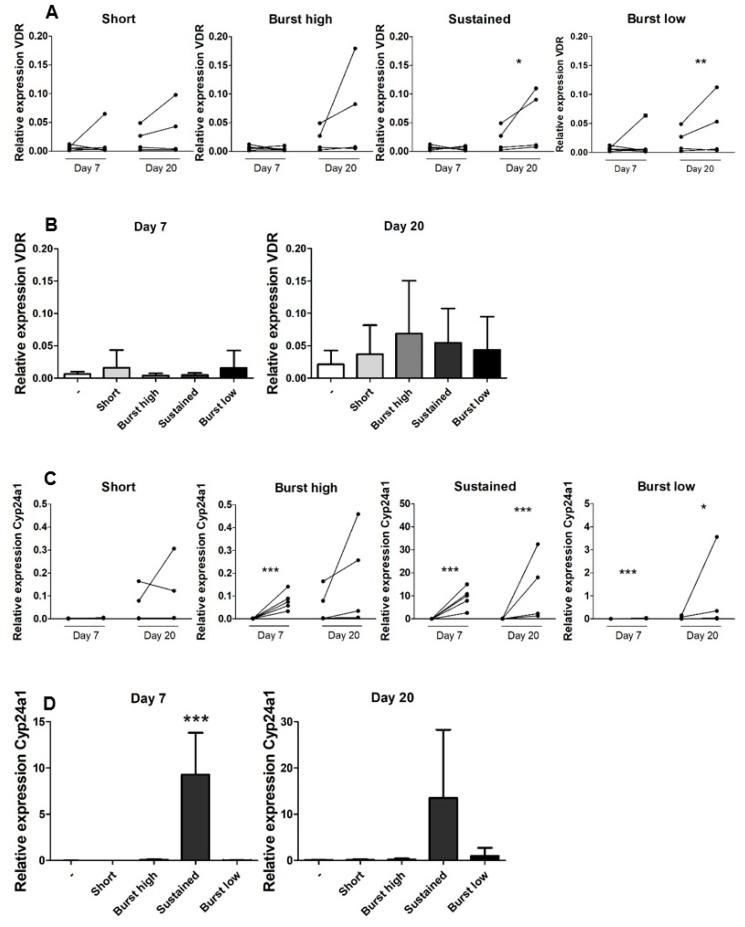
Gene expression of *VDR* and *CYP24a1***.** (**A**) *VDR* expression. Data of controls (left) and vitD_3_ treated cells (right) from the same donor are connected with a line. (**B**) Average relative expression of *VDR* at Days 7 and 20. Data represent four or five independent donors (mean + SD). (**C**) Burst high, Sustained, and Burst low application of vitD_3_ significantly increased *CYP24a1* (note the 10-fold differences in axis scale). Data of controls (left) and vitD_3_ treated cells (right) from the same donor are connected with a line. (**D**) Average relative expression of *CYP24a1* at Days 7 and 20. Data represent four or five independent donors (mean + SD). All gene expression data were normalized to *HPRT*. (A,C) * Significant difference (*p* < 0.05) between control and vitD_3_ treatment at the same time point; *** *p* < 0.001. (D) * Significant difference (*p* < 0.001) between Sustained vitD_3_ treatment and all other groups.

To analyze whether vitD_3_ stimulates a feedback loop causing insensitivity of the cells for vitD_3_ and inactivation of the vitD_3_ itself [26], the expression of vitamin D receptor (*VDR*) and *CYP24a1* was measured. *VDR* was expressed at low levels on Day 7, regardless of the addition of vitD_3_. At Day 20, vitD_3_ seemed to have an overall stimulating, rather than inhibiting, effect on the expression of *VDR*. The stimulating effect of vitD_3_ on *VDR* expression by hASCs was significant for Sustained and Burst low applications (Figure 5A). Average expression levels of *VDR* did not significantly differ between groups (Figure 5B). *CYP24a1* converts the active form of vitD_3_ in Calcitroic acid, an inactive vitamin D metabolite [26]. All vitD_3_ applications, except for the condition “Short”, enhanced the expression of *CYP24a1* compared to the control condition at Day 7. At Day 20, Sustained and Burst low significantly increased *CYP24a1* compared to the control (Figure 5C). At Days 7 and 20 Sustained vitD_3_ application strongly upregulated average expression levels of *CYP24a1* compared to all other groups (Figure 5D).

## 3. Discussion

In this study, we evaluated which vitD_3_ application (Short, Burst, or Sustained) to hASCs seeded on BCP particles, leads to optimal osteogenic differentiation of hASCs in cell culture. We did this because we wished to mimic the effect of different release kinetics of vitD_3_ from biomaterials in vivo. Our results may help to design scaffolds with bioactive components for bone tissue engineering in a more targeted manner, and suggest that applying a daily dose of vitD_3_ in vitro (Sustained application), thereby mimicking a sustained release of vitD_3_ in vivo, stimulates ALP activity of hASCs, where other applications do not, even when the same total amount of vitD_3_ (124 ng) was applied.

In this study, we mimicked short, burst, and sustained release from a scaffold. The same total amount of vitD_3_ was used for short, burst high, and Sustained applications (i.e., 124 ng), but delivered over different time spans, thereby leading to different concentrations. The concentration of 10 nM thus obtained for the Sustained application is a widely used concentration to induce osteogenic differentiation of MSCs [7,25,27]. Adding the same 124 ng vitD_3_ in two days (Burst high) or 30 min (Short) led to concentrations of 100 and 200 nM respectively. For Burst low, we applied the same concentration of vitD_3_ as the Sustained application (10 nM), but adhered to the same two-day timeframe as burst high, which means that the total amount of vitD_3_ added to the ASCs over the total culture period was only 6.4 ng.

Our method of mimicking release from scaffolds has a limitation. The medium was refreshed every day to mimic a steady sustained release of bioactive components more accurately. This means the cells got a “shock” from the medium refreshment every day. This might have influenced the responses of the cells to vitD_3_ compared to more regular treatment regiments, where it is customary that cells are replenished two or three times a week. It could explain our relative lack of differentiation induced by vitD_3_, where we did not observe an obvious effect of vitD_3_ on ASC differentiation. Despite this downside, we were still able to discern clear effects of vitD_3_ on *RUNX2*, *VDR*, and *CYP24* expression and ALP activity, and the cells used in the present study proliferated as normal (Figure 3). If our results are indeed somewhat “blunted” by the daily medium refreshments, it is to be expected that the observed differences of our in vitro investigation are *more* pronounced in vivo. In addition, the ASCs seemed to show an increased *BCL-2* expression over time, while *BAX* expression remained level, indicating that the frequent medium refreshments did not induce apoptosis. Importantly, since all our groups were treated in exactly the same manner, we can readily compare the effects of our vitD_3_ applications between groups.

One of the first prerequisites for successful tissue engineering approaches is the availability of sufficient numbers of cells. Cell number increases through proliferation or decreases through cell death. VitD_3_ (1–100 nM) shows an inverse linear relation with proliferation in human osteoblasts and C_2_C_12_ cells [28,29]. Whether vitD_3_ decreases proliferation in hASCs is unknown. A similar inhibition of proliferation of hASCs by vitD_3_ could have adverse effects on bone healing by limiting available cell numbers, unless perhaps the reduction in proliferation is explained by a strong stimulation of differentiation. Unfortunately, the use of calcium-containing biomaterials did not permit us to use DNA as a measure for cell number, but we used the following surrogates for cell number: the Alamar blue assay (Figure 3A; which unfortunately also takes metabolic activity into account), histology (Figure 2A–D), and total protein (Figure 3C). In Figure 3C, it is clearly visible that the total protein content is highly similar between groups at Day 7. This is supported by the Alamar blue data from Day 2 and 7, which show no differences. Based on these data, and the results from histology from Day 2, we can assume that the initial seeding of the particles was homogeneous. Our Alamar blue results, in combination with protein measurements, show that cell number varied very little between vitD_3_-treated conditions. Together, this indicates that the different vitD_3_ applications (Sustained (10 nM), Burst high (100 nM), Burst low (10 nM), or Short (200 nM)) neither reduced nor enhanced proliferation of ASCs (Figure 3). This was confirmed by gene expression of *Ki67*, a marker for proliferation, which also showed no reduction.

Cell number can be affected by cell death, through necrosis or apoptosis. In large tissue engineering constructs, low oxygen tension in the center of the construct can lead to rapid loss of stem cells. Notably, vitD_3_ can cause apoptosis in mouse adipocytes (3T3-L1) at a concentration of 77.4 nM ± 23.20 at Day 3 and 61.7 nM ± 20.5 at Day 6 [30] and breast cancer cells (MCF-7) at a concentration of 100 nM [31]. The results of the live-dead staining at Day 2 shows that even the highest concentration of vitD_3_ (Short, 200 nM) did not cause immediate cell death of hASCs. However, this does not guarantee that vitD_3_ does not induce cell death at later time points. We therefore quantified gene expression of *BAX* and *BCL-2* as a measure for apoptosis. Interestingly, the relative expression of *BCL-2*, an inhibitor of pro-apoptotic proteins, seemed to increase at Day 20 compared to Day 7 (not tested), regardless of whether vitD_3_ was added, which suggests that inhibition of pro-apoptotic proteins increased over culture time. This general increase in BCL-2 has also been observed in human osteoblasts cultured on plastic with or without a treatment of 10 nM vitD_3_ [32,33]. By Day 7, the gene expression of *BAX*, a pro-apoptotic molecule, had been upregulated in the condition mimicking sustained release (10 nM per day for 20 days) and, by Day 20, the expression of *BAX* was upregulated by the condition mimicking burst release (100 nM per day for two days; Figure 2E). The increase in *BAX* expression in these conditions may indicate initiation of apoptosis, but this also depends on the expression of *BCL-2*. Gene expression of *BCL-2* was not significantly increased for any of the vitD_3_ treatments. Interestingly, total protein and Alamar blue did not show a decrease for Sustained and Burst high at any time point (Figure 3), suggesting that, if increased *BAX* expression was indeed translated into an increased balance between *BAX* and *BCL-2* protein, then it the impact on hASC apoptosis was minor. Our results together show that none of our vitD_3_ applications seems harmful for hASCs.

Against expectations, expression of the early osteogenic marker *RUNX2* appeared to be higher at Day 20 than at Day 7, in both the presence and absence of vitD_3_. This trend was not statistically tested, but was striking, as the same pattern was observed for all treatment groups. As it has already been shown that calcium phosphate materials can induce the upregulation of *RUNX2* [34] and that Ca^2+^ ions can play a role in the differentiation of stem cells/osteoblasts [35], we suspect that the apparent upregulation of *RUNX2* over time is due to the BCP particles, which likely released ions in the medium, as they are known to do [35,36]. Burst high and Sustained application of vitD_3_ significantly reduced *RUNX2* expression. This is in line with other studies [37,38] However, the average gene expression levels of *RUNX2* did not significantly differ between treatment groups, suggesting that Burst high and Sustained had no superior effect on hASC differentiation with respect to *RUNX2* expression.

Our results suggest that for the osteogenic differentiation of adipose stem cells cultured in 3D in vitro, continuous exposure to vitD_3_ or “Sustained release” might be preferable compared to shorter applications, but we are extremely careful with this conclusion, as it is based on ALP activity only. *OPN* expression was unaffected by vitD_3_ application, and, due to the use of BCP particles (containing calcium and phosphate), it was not possible to perform analyses such as alizarin red staining. However, at Days 7 and 20, we found that the early osteogenic marker ALP had been significantly increased by the Sustained application. Mokhtari-Jafari et al. showed that a short (30 min) incubation of hASCs with vitD_3_ led to higher ALP expression than sustained incubation [25]. The contradictions between their findings and ours might be explained by a difference in concentration of the 30-min pre-incubation, as we used a 20× higher concentration. Although the concentration they used for the sustained application is the same as in this study, the total amount of vitD_3_ sensed by our cells is higher, due to daily rather than twice-weekly refreshment of the medium. This indicates that for short incubation, the concentration of vitD_3_ may be important for the osteogenic differentiation of hASCS, while for sustained application the total amount applied may play a role as well, at least in vitro. This information could be valuable for the development of scaffolds releasing bioactive components, assuming that our results translate to the in vivo situation.

A potential problem with application of high amounts of active factors, or long durations of application of such factors, is that cells may respond less to further stimulation by those factors, through altered expression of receptors or inhibitors. *VDR* is the main receptor through which vitD_3_ regulates biological responses [36]. The expression of *VDR* in this study was quite stable, and, overall, the expression was not downregulated, indicating that the various vitD_3_ applications as used in the current study did not make the cells become less sensitive for vitD_3_. *CYP24a1* converts the active vitD_3_ to an inactive form [39] and in our study it was highly upregulated by Sustained application of vitD_3_. The rapid inactivation of vitD_3_ may have reduced the effect of Sustained vitD_3_ application on hASC differentiation, thereby explaining why the early osteogenic differentiation marker ALP was still upregulated at Day 20. On the other hand, ALP might still be upregulated at Day 20 because of the 3D culture system we used. ALP is an early marker when cells are cultured on plastic, but cultured in 3D the ALP expression increases slowly over time [40,41,42]. For future research, it is interesting to mimic a sustained release with even lower concentrations, since we speculate that this will decrease the inactivation of vitD_3_ by *CYP24a1*.

Sustained application stimulated ALP activity, where other applications of vitD_3_ did not, without inhibition of proliferation or reduction in cell number. This suggests that tissue engineering solutions in which an active compound such as vitD3 is released over a long period of time may be more beneficial for stimulating osteogenic differentiation than shorter durations of application. In line with these results, Faßbender et al. showed that sustained release of a low amount of BMP-2 had a similar positive effect on bone healing as higher BMP-2 amounts released with a burst [43]. On the other hand, a short application—if it works—can be clinically relevant as no exogenous ligands are placed in the body, thereby preventing potential adverse side effects. In the case of vitD_3_, this could be osteoclastogenesis. VitD_3_ can induce *RANKL*, which can induce the formation of osteoclasts as shown in a murine monocyte/macrophage cell line at doses as low as 10 nM, which is comparable to the concentration we used in the sustained application [44]. We do not expect these kind of adverse effects of sustained release from a biomaterial at lower concentrations, yet each patient reacts differently, and factors such as race and gender may affect the response to vitD_3_, therefore patient specific treatment designs might be a good option to consider [45]. The clear donor to donor variability in the response to vitD_3_ treatment is visible in our data. This is most visible in the qPCR data (Figure 4 and Figure 5) where in some cases the effect of vitD_3_ on gene expression is higher in two out of the five donors. The race of our donors is unknown, which is unfortunate, as 25-hydroxyvitamin D_3_ serum levels and *VDR* expression vary between white and black people, possibly affecting the response to vitD_3_ [46]. Importantly, the donors that respond strong to viD_3_ differ from gene to gene analysis, and there are not two individuals that can be designated as “high responders” vs. “low responders”. It is theoretically possible that hASCs from male donors react differently than those of females, but in our study at least four out of five of our donors are female.

In summary, although the effects of vitD_3_ on osteogenic differentiation were limited to ALP activity and *RUNX2* expression, the results of this present study are still convincing enough to prompt us to reject our hypothesis that a short incubation with vitD_3_ would be more beneficial for osteogenic differentiation than other durations of applications. Adding vitD_3_ for a longer period did not lead to apoptosis, and induced the ALP expression at both Days 7 and 20. In addition, it affected gene expression of *RUNX2*, *VDR*, and *CYP24a1*. Short application did none of these things. Overall, a Sustained application seems to have more effect on hASCs compared to shorter applications, and therefore it would be wise to further investigate the effect of sustained release of vitamin D_3_ on proliferation and osteogenic differentiation of stem cells, taking into account factors such as donor-to-donor variability, broad dose-response studies, and possible side effects.

## 4. Materials and Methods

The release of vitD_3_ from scaffolds was mimicked by exposing the hASCs seeded on a 3D biomaterial to controlled vitD_3_ applications, and the viability, proliferation, and differentiation were measured.

### 4.1. Calcium Phosphate Particles

Clinically relevant, biphasic calcium phosphate (BCP, Straumann^®^ BoneCeramic 60/40, Institut Straumann AG, Basel, Switzerland) particles with a particle size of 500–1000 µm and a porosity of 90% were used to mimic calcium phosphate containing scaffolds in vitro.

### 4.2. Isolation of hASCs

For the isolation of hASCs, subcutaneous adipose tissue from the abdominal wall was harvested from donors who underwent plastic surgery at the Tergooi Hospital Hilversum, the Netherlands. The study was conducted in accordance with the Declaration of Helsinki and the protocol was approved by the Ethical Review Board of the VU Medical Center, Amsterdam, the Netherlands (number 2016/105). Informed consent was obtained from all donors. Four of the donors were female and between 33 and 56 years of age. Information on the fifth donor is unavailable.

To isolate hASCS, adipose tissue was cut into pieces and digested enzymatically with 0.1% collagenase A (Roche Diagnostics GmbH, Mannheim, Germany) which was added to phosphate-buffered saline (PBS; Thermo Fisher Scientific, Waltham, MA, USA) containing 1% bovine serum albumin (Roche Diagnostics GmbH) under stirring conditions for 45 min at 37 °C. This was followed by a Ficoll^®^ density-centrifugation step (Lymphoprep; 1000× *g*, 20 min, ⍴ = 1.077 g/mL Ficoll^®^, osmolarity 280 ± 15 mOsm; Axis-Shield, Oslo, Norway) and the cell-containing interface was harvested and resuspended in Dulbecco’s modified Eagle’s medium (Life Technologies Europe BV, Bleiswijk, the Netherlands). hASCS were counted and stored in liquid nitrogen [24]. All experiments were conducted using the same five donors, but sometimes data from only four donors were available.

### 4.3. Platelet Lysate

Bloodbank Sanquin (Sanquin, Amsterdam, the Netherlands) provided pooled platelet products from multiple donors, which contained approximately 1 × 10^9^ platelets per mL PAS-E/plasma (ratio 65:35) [47]. Platelets were lysed by a temperature shock at ‒80 °C to obtain platelet lysate (PL). Before use, PL was thawed and centrifuged at 600× *g* for 10 min to remove remaining platelet fragments. Finally, the supernatant was added to the medium at 5% (*v*/*v*) for the culturing of cells and 2% (*v*/*v*) for the experiments [48]. PL was used as a human cell culture supplement rather than bovine serum.

### 4.4. Culture and Treatment of hASCs

hASCs (1 × 10^5^) from five independent donors were seeded, separately, on top of 20 ± 1 mg BCP particles in 2 mL Eppendorf tubes. Before seeding, the BCP particles were soaked in 500 µL of Minimum Essential Medium ALPHA (α-MEM; Thermo Fisher Scientific) complemented with 1% Antibiotic Antimycotic Solution 100× (Sigma, St Louis, MO, USA), 10 IU/mL heparin (Leo Pharma, Amsterdam, the Netherlands), 2% PL, and 50 µM ascorbic acid-2-phosphate (vitamin C; Sigma) for 30 min. For the short stimulation, the hASCs were treated with 124 ng of vitD_3_ for 30 min ([200 nM]), before seeding. Then, cells (all treatments) were allowed to attach to the BCP particles for 1 h at 37 °C and transferred to 12 well plates with transwell inserts (pore size 3.0 µm; Greiner Bio-one, Alphen aan de Rijn, the Netherlands) with 0.5 mL of medium in the insert and 1 mL of medium in the well. For Burst and Sustained release this medium was supplemented with the total amount of 124 ng of vitD_3_ (Sigma, stock: 30 µM in 100% ethanol, stored at -80 °C, in the absence of light) per well. Control cultures contained the same concentration of alcohol as in the sustained condition (3.35 µL 100% alcohol per 10 mL of medium). The vitD_3_ treatment was provided either over the first two days (Burst-release high: [100 nM]), or over the total culture period of 20 days (sustained-release: [10 nM]). In the condition “Burst-release low” (Burst low), the hASCs were treated for two days after seeding with 6.2 ng vitD_3_ ([10 nM]) per day. A schematic representation of the applications is depicted in Figure 1. Medium of all conditions was refreshed daily.

### 4.5. Attachment and Viability

Attachment and viability were assessed using fluorescent microscopy. After two days, inserts were emptied, by cutting the membrane of the insert, in a clean 12-wells plate. Medium was removed and the BCP particles with cells were washed once with PBS and incubated for 10 min with 5× live/dead staining (Abcam, Cambridge, UK) in PBS. The dye was removed and 100 µL of PBS was added to the samples. The staining was analyzed with a fluorescence microscope (Leica Microsystems, Wetzlar, Germany) with a Nikon camera (Nikon, Tokyo, Japan). Brightness and contrast were adapted to enhance the visibility of the cells with Adobe Photoshop^®^ 19.1.1 (Adobe Systems, San Jose, CA, USA). The number of live (green) and dead (red) cells were manually counted, bt due to the difficulty of discerning individual cells, only qualitative assessments were made.

### 4.6. Metabolic Activity

Metabolic activity of hASCs was measured with AlamarBlue™ Cell viability reagent (Invitrogen, Carlsbad, CA, USA). Metabolic activity was measured at Days 2, 7, and 20. Inserts with BCP particles were placed in a new set of wells and incubated for 4 h at 37 °C with medium containing 10% AlamarBlue reagent (500 µL in the well and 500 µL in the insert). After incubation, the medium in the insert was mixed with the medium in the well. From each sample, 100 µL (in duplicate) were transferred to a black 96-well plate and fluorescence was measured at 530–560 nm with Synergy HT^®^ spectrophotometer (BioTek Instruments, Winooski, VT, USA). Medium without cells and with 10% AlamarBlue reagent was autoclaved to fully reduce the AlamarBlue reagent and used as a positive control. Samples are presented as percentage of the positive control.

### 4.7. Alkaline Phosphatase (ALP)

hASCs were cultured on BCP particles for 7 and 20 days with the different vitD_3_ applications or without vitD_3_. Cells were washed with PBS and lysed with 300 µL Milli-Q water and frozen in ‒20 °C for storage. After three freeze–thaw cycles, samples were collected by scraping. ALP was measured according to the method described by Lowry [49] using 4-nitrophenyl phosphate disodium salt at pH 10.3 as a substrate for ALP. Absorbance was measured at 405 nm with the Synergy HT^®^ spectrophotometer. To correct for cell number, protein levels were measured using a bicinchoninic acid (BCA) Protein Assay Kit according to the manufacturers’ instructions (Pierce, Rockford, IL, USA) and absorbance was read at 540 nm with Synergy HT^®^ spectrophotometer. ALP was expressed as nmol/µg protein.

### 4.8. Quantitative Polymerase CHAIN reaction (qPCR)

After 7 and 20 days of culture, the inserts with the BCP particles were transferred to a clean 12-wells plate. Total RNA was isolated with TRIzol^®^ reagent (Invitrogen) following the manufacturer’s protocol. The concentration and 260/280 ratio of RNA were measured using a Synergy HT^®^ spectrophotometer and 750 ng RNA was reverse-transcribed to cDNA using RevertAid™ First Strand cDNA Synthesis Kit 1612 (Fermentas, St. Leon-Rot, Germany) according to the manufacturer’s protocol. For the qPCR reaction, cDNA was diluted 5× and 1 µL was used, together with 3 µL PCR-H_2_O, 0.5 µL (20 µM) forward primer, 0.5 µL (20 µM) reverse primer, and 5 µL LightCycler^®^ 480 SYBR Green I Mastermix (Roche Diagnostics). All measurements with a Ct value higher than 36 were considered unreliable and discarded. The values of all genes were normalized to hypoxanthine phosphoribosyl transferase (HPRT) following the comparative cycle threshold (*C*_t_) method and presented as the mean relative fold expression (2^−Δ*C*t^). All primer sequences are listed in Table 1.

### 4.9. Statistical Analysis

Data were obtained from cultures of five independent donors (*n* = 5), performed in duplicate, and are presented as mean ± standard deviation (SD). Since the *n* was small, and the data are not normally distributed, the data were transformed with a log transformation. A paired t-test was conducted to test for statistical differences in gene expression levels between control and vitD_3_ applications per time point and treatment, for results depicted as single point graphs. Statistical comparisons of the treatment groups (short, burst high, burst low, and sustained) in Alamar blue, protein content, ALP activity, and average gene expression levels were performed using repeated-measures ANOVA with Tukey’s post hoc test. *P* values of <0.05 were considered significantly different. Analyses were performed using GraphPad Prism 5.0 (GraphPad Software, San Diego, CA, USA).

## Figures and Tables

**Table 1:** Primer sequences.

Gene (Human)	Forward Sequence	Reverse Sequence
*KI67*	5′ CGAGACGCCTGGTTACTATCAA 3′	5′ GGATACGGATGTCACATTCAATACC 3′
*RUNX2*	5′ CCAGAAGGCACAGACAGAAGCT 3′	5′ AGGAATGCGCCCTAAATCACT 3′
*COL1*	5′ GCATGGGCAGAGGTATAATG 3′	5′ GGTCCTTTGGGTCCTACAA 3′
*BAX*	5′ TGTCGCCCTTTTCTACTTTGC 3′	5′ CTGATCAGTTCCGGCACCTT 3′
*BCL-2*	5′ AGAGCCTTGGATCCAGGAGAA 3′	5′ GCTGCATTGTTCCCATAGAGTTC 3′
*VDR*	5′ GACACAGCCTGGAGCTGAT 3′	5′ CAGGTCGGCTAGCTTCTGGA 3′
*CYP24a1*	5′ CAAACCGTGGAAGGCCTATC 3′	5′ AGTCTTCCCCTTCCAGGATCA 3′
*HPRT*	5′ GCTGACCTGCTGGATTACAT 3′	5′ CTTGCGACCTTGACCATCT 3′
*OPN*	5′ TTCCAAGTAAGTCCAACGAAAG 3′	5′ GTGACCAGTTCATCAGATTCAT 3′
*PPAR- γ*	5′ CGACCAGCTGAATCCAGAGT 3′	5′ GATGCGGATGGCCACCTCTT 3′

## Supplementary Materials

Supplementary materials can be found at https://www.mdpi.com/1422-0067/21/9/3202/s1.

## Abbreviations

vitD_3_	1,25(OH)2 vitamin D_3_
MSCs	mesenchymal stem cells
hASCs	human adipose stem cells
ALP	alkaline phosphatase
OPN	osteopontin
BCP	biphasic calcium phosphate
BMP-2	bone morphogenetic protein-2
VEGF	vascular endothelial growth factor
BMSCs	bone marrow-derived mesenchymal stem cells
CYP24a1	Cytochrome P450 family 24 subfamily A member 1
BAX	B-cell lymphoma protein 2 associated X
BCL-2	B-cell lymphoma 2
RUNX2	Runt-related transcription factor 2
COL1a	collagen type 1a
PPAR-γ VDR	Peroxisome proliferator-activated receptor gammavitamin D receptor
qPCR	Quantitative polymerase chain reaction
HPRT	hypoxanthine phosphoribosyl transferase
