# Supplementary material for: Burst, Short, and Sustained Vitamin D3 Applications Differentially Affect Osteogenic Differentiation of Human Adipose Stem Cells
Source: Int J Mol Sci. 2020 Apr 30;21(9):3202. doi: 10.3390/ijms21093202 (PMC7247321; doi:10.3390/ijms21093202)
Supplement: Supplementary file 1 [file ijms-21-03202-s001.pdf]

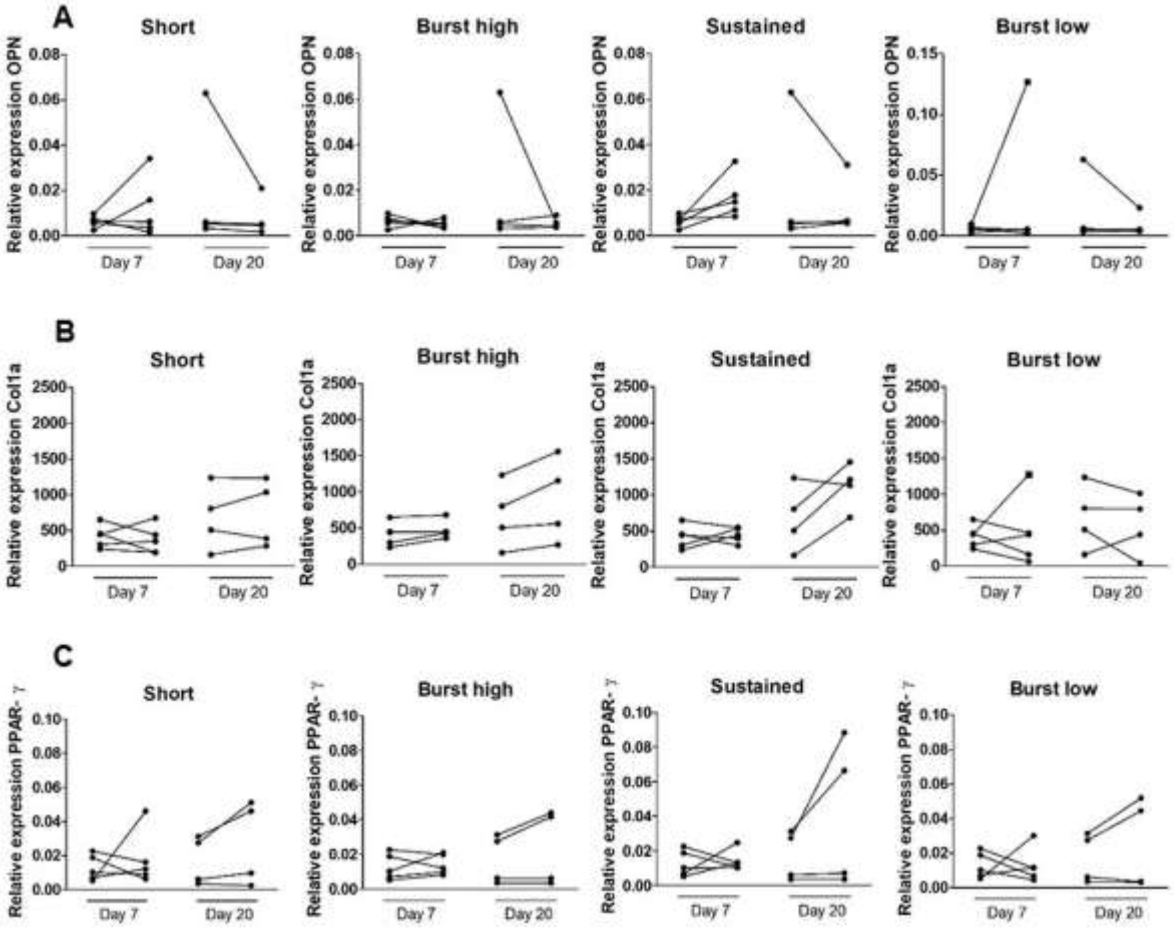

Figure S1. (**A**) Gene expression of the osteogenic marker OPN measured at day 7 and day 20. (**B**) Gene expression of COL1a1 was measured at day 7 and day 20. (**C**) Gene expression of the adipogenic marker PPARγ was measured at day 7 and day 20. vitD$_3$ did not affect the expression of any of these markers. Gene expression in controls (left) and vitD$_3$-treated cultures (right) of the same donor are connected by a line. All gene expression levels were normalized to HPRT.
